# Hope-inspiring competence as a high-quality mental health nursing care in recovery-oriented practice

**DOI:** 10.1192/j.eurpsy.2022.2263

**Published:** 2022-09-01

**Authors:** C. Laranjeira, A. Querido

**Affiliations:** Polytechnic of Leiria, School Of Health Sciences/ Citechcare, Leiria, Portugal

**Keywords:** Recovery, Hope, mental health nursing

## Abstract

**Introduction:**

Hope should be fostered by providing information to help service users develop an understanding of psychological difficulties and encourage an active role in their self-care. This might include providing an open caring environment, nurse presence, comfort/pain relief, and involving patients in their care.

**Objectives:**

To analyze the critical importance of hope-inspiring competence as a high-quality mental health nursing care in recovery-oriented practice.

**Methods:**

This was a reflective and discursive study based on experiential aspects of hope in mental health recovery.

**Results:**

Hope and hopelessness are important determinants of mental health. Hope has a positive influence on people’s mental health, on increasing comfort and satisfaction with life and on reducing negative emotions and suicide, decreasing the predisposition to addiction, and preventing family exhaustion, with a predictive effect on subjective well-being and protection of mental health. The concept of hope-inspiring competence is introduced to denote a relatively high level of the Advanced Practice Psychiatric Nurses ability to instil and maintain hope for recovery in people with mental health disorders.

**Conclusions:**

The evidence seems to point to the importance of incorporating hope in collaborative strategies to promote mental health and manage mental health disorders. Despite this recognition of the role of the specialist nurse in psychiatric-mental health in training for hope, the way it develops in the context of specialized practice lacks evidence and visibility. A vision of recovery from mental illness exists, and hope, trust and self-determination should be incorporated into all treatment models.

**Disclosure:**

No significant relationships.

